# Conserved Central Intraviral Protein Interactome of the *Herpesviridae* Family

**DOI:** 10.1128/mSystems.00295-19

**Published:** 2019-10-01

**Authors:** Anna Hernández Durán, Kay Grünewald, Maya Topf

**Affiliations:** a Institute of Structural and Molecular Biology, Birkbeck College, University of London, London, United Kingdom; b Division of Structural Biology, Wellcome Centre for Human Genetics, University of Oxford, Oxford, United Kingdom; c Department of Structural Cell Biology of Viruses, Centre for Structural Systems Biology, Heinrich Pette Institute, Leibnitz Institute of Experimental Virology, University of Hamburg, Hamburg, Germany; National Institutes of Health

**Keywords:** herpesviruses, intraviral network, protein-protein interactions, systems biology

## Abstract

Protein interactions are major driving forces behind the functional phenotypes of biological processes. As such, evolutionary footprints are reflected in system-level collections of protein-protein interactions (PPIs), i.e., protein interactomes. We conducted a comparative analysis of intraviral protein interactomes for representative species of each of the three subfamilies of herpesviruses (herpes simplex virus 1, human cytomegalovirus, and Epstein-Barr virus), which are highly prevalent etiologic agents of important human diseases. The intraviral interactomes were reconstructed by combining experimentally supported and computationally predicted protein-protein interactions. Using cross-species network comparison, we then identified family-wise conserved interactions and protein complexes, which we defined as a herpesviral “central” intraviral protein interactome. A large number of widely accepted conserved herpesviral protein complexes are present in this central intraviral interactome, encouragingly supporting the biological coherence of our results. Importantly, these protein complexes represent most, if not all, of the essential steps required during a productive life cycle. Hence the central intraviral protein interactome could plausibly represent a minimal infectious interactome of the herpesvirus family across a variety of hosts. Our data, which have been integrated into our herpesvirus interactomics database, HVint2.0, could assist in creating comprehensive system-level computational models of this viral lineage.

**IMPORTANCE** Herpesviruses are an important socioeconomic burden for both humans and livestock. Throughout their long evolutionary history, individual herpesvirus species have developed remarkable host specificity, while collectively the *Herpesviridae* family has evolved to infect a large variety of eukaryotic hosts. The development of approaches to fight herpesvirus infections has been hampered by the complexity of herpesviruses’ genomes, proteomes, and structural features. The data and insights generated by our study add to the understanding of the functional organization of herpesvirus-encoded proteins, specifically of family-wise conserved features defining essential components required for a productive infectious cycle across different hosts, which can contribute toward the conceptualization of antiherpetic infection strategies with an effect on a broader range of target species. All of the generated data have been made freely available through our HVint2.0 database, a dedicated resource of curated herpesvirus interactomics purposely created to promote and assist future studies in the field.

## Abstract (precis)

Herpesviruses are an important socioeconomic burden for both humans and livestock. Throughout their long evolutionary history, individual herpesvirus species have developed remarkable host specificity, while collectively the *Herpesviridae* family has evolved to infect a large variety of eukaryotic hosts. The development of approaches to fight herpesvirus infections has been hampered by the complexity of herpesviruses’ genomes, proteomes, and structural features. The data and insights generated by our study add to the understanding of the functional organization of herpesvirus-encoded proteins, specifically of family-wise conserved features defining essential components required for a productive infectious cycle across different hosts, which can contribute toward the conceptualization of antiherpetic infection strategies with an effect on a broader range of target species. All of the generated data have been made freely available through our HVint2.0 database, a dedicated resource of curated herpesvirus interactomics purposely created to promote and assist future studies in the field.

## INTRODUCTION

Herpetic infections are ubiquitous and highly prevalent that affect a wide range of eukaryotic organisms. In humans and livestock, they are often associated with mild symptoms, yet they can develop into severe conditions through the lack of appropriate treatment or a weak host immune response. Serious conditions associated with these diseases include encephalitis, neurosensory birth defects, and several types of cancer (1). As a consequence, they represent an important socioeconomic burden (2–4). Despite the wide range of organisms that herpesviruses, as a family, infect, each species tends to show a high host specificity. This is the result of millions of years of subtly tuned coevolution with their respective hosts (1).

The human herpesviruses are a subgroup of currently nine known herpesvirus species that recurrently infect humans as their natural host and with which they are estimated to have been coevolving for over 400 million years (5). The nine species are found across the three subfamilies in which the *Herpesviridae* family is divided. Herpes simplex virus 1 (HSV-1, or human alphaherpesvirus 1 [HHV-1]), HSV-2 (or HHV-2), and varicella-zoster virus (VZV, or HHV-3) are members of the *Alphaherpesvirinae* subfamily. Human cytomegalovirus (HCMV, or human betaherpesvirus 5 [HHV-5]), human betaherpesviruses 6A and 6B (HHV-6A and HHV-6B, respectively), and human betaherpesvirus 7 (HHV-7) belong to the *Betaherpesvirinae* subfamily. Epstein-Barr virus (EBV, or human gammaherpesvirus 4 [HHV-4]) and Kaposi’s sarcoma-associated herpesvirus (KSHV, or human gammaherpesvirus 8 [HHV-8]) are part of the *Gammaherpesvirinae* subfamily. These nine species show different cell tropisms, and their infections result in distinct symptomatologies (1). As in other organisms, the great majority of these phenotypical features are not mediated by the product of one single gene but a number of them and, importantly, their physical and functional interactions. As a consequence, intraspecies interactions among virally encoded proteins have attracted interest over the years (6–9). However, comprehensive nonredundant compilations of these data are still largely missing.

Previously, we developed HVint (10), a centralized resource of curated protein-protein interaction (PPI) data for HSV-1, the prototypical species among human herpesviruses. The development of an improved bioinformatics protocol for PPI network inference led to the construction of our new database, HVint2.0, also dedicated to PPIs in HSV-1 (11). Here, we introduce the extension of HVint2.0 to contain curated PPI data across representative species of the *Herpesviridae* family. To this end, we applied our recently developed bioinformatics PPI network reconstruction pipeline (11) to generate PPIs for HCMV and EBV. Taking advantage of the family-wise representation of PPI networks, we inferred a conserved “central” interactome of the *Herpesviridae* family. This reconstructed central interactome portrays a large number of well-known conserved protein complexes within the family, which we deem indicative of its biological coherence. Coupling the information extracted from subfamily-specific intraviral networks and the central interactome could become a useful tool to assist experimental interactomics studies and reveal new insightful knowledge on the evolutionary dynamics of the family and identify system-level patterns that can explain some of the different phenotypical features observed across species.

## RESULTS

### HVint2.0 database.

**FIG 1:**
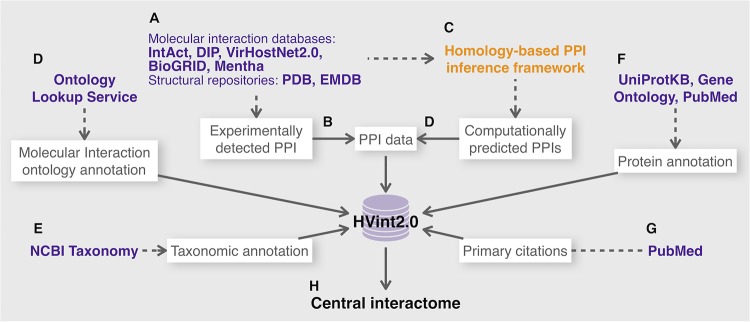
HVint2.0 data integration framework. Blue text indicates external resources (e.g., databases and Web servers), orange indicates data transformations (e.g., interaction prediction from raw input data), and text in white boxes refers to the data sets collated from these external resources. (A) Experimentally supported PPI evidence is collated from public repositories. (B to D) Experimentally supported PPIs are used as direct PPI input to HVint2.0 and to infer further PPIs based on sequence homology, which are then further introduced into HVint2.0. (F and G) From the resulting curated compilations, all corresponding unique identifiers for PSI-MI annotation codes, taxonomic ranks, protein species, and primary citations are collected, and their corresponding annotation data are included in HVint2.0. (H) The family-wise PPI data contained in HVint2.0 were used in this study to generate a candidate family-wise conserved interactome.

To populate the HVint2.0 database (Fig. 1; see Fig. S1 in the supplemental material), we generated curated compilations of PPI data for human herpesviruses HSV-1, HCMV, and EBV, representative members of the *Alpha-*, *Beta-*, and *Gammaherpesvirinae* subfamilies, respectively, using our recently described computational network reconstruction framework (11). In brief, our framework (see Materials and Methods and see Fig. S2 in the supplemental material) integrates, for each of these three (target) species, experimentally obtained evidence and computational PPI predictions (inferred using a conservative sequence similarity-based approach). The framework also integrates a refined scoring function that assesses the confidence of each interaction, taking into account the heterogeneity of its cumulative supporting evidence. The heterogeneity present among experimentally obtained supporting evidence is accounted for using the MIscore function (12). For those interactions that are computationally inferred using homology, we include an additional penalizing scaling factor, which is dependent on the family-wise conservation of the PPI: i.e., the wider the conservation of a PPI across the family, the higher the confidence score of the prediction. Below, for each reconstructed network, experimentally supported PPIs are referred to as “ePPIs,” computationally predicted ones as “pPPIs,” and those PPIs that had both computational and experimental support as ePPIs *∩* pPPIs.

10.1128/mSystems.00295-19.2 FIG S1 Physical schema of the HVint2.0 database. The data in HVint2.0 are organized into seven tables: i.e., “ppi,” “protein,” “evidence,” “evidence_to_psimi,” “psimi_ontology,” “taxon,” and “citation.” The content of each of table is detailed in the accompanying portion of Text S1. For each table, the figure displays the table name and the list of columns in the table, together with their data type. Primary keys are indicated with a key icon next to the corresponding column name; columns that can and cannot hold null values are indicated with empty and filled rhomboid icons, respectively. Download FIG S1, TIF file, 0.5 MB. Copyright © 2019 Hernández Durán et al. 2019 Hernández Durán et al. This content is distributed under the terms of the Creative Commons Attribution 4.0 International license.

10.1128/mSystems.00295-19.3 FIG S2 Network reconstruction framework. (A) PPI data are collected from several public data resources and for the taxons of interest. These data include PPIs in the target taxon and in a number of orthologous species. (B) PPIs detected in orthologous species are the input for stage, where sequence-based homology assignments are used to predict new PPIs in the target taxon (green enlarged frame). (C) Predicted PPIs (pPPIs) and PPIs originally detected in the target taxon present in the input data set (tPPIs) are nonredundantly integrated in the final network. Each PPI in the final network is scored under the same confidence scoring scheme. (D) This framework was applied, separately, for each of the three representative target species of human herpesviruses (HSV-1, HCMV, and EBV) to reconstruct a corresponding network. Download FIG S2, TIF file, 0.7 MB. Copyright © 2019 Hernández Durán et al. 2019 Hernández Durán et al. This content is distributed under the terms of the Creative Commons Attribution 4.0 International license.

10.1128/mSystems.00295-19.1 TEXT S1 Database structure and content. Download Text S1, DOCX file, 0.02 MB. Copyright © 2019 Hernández Durán et al. 2019 Hernández Durán et al. This content is distributed under the terms of the Creative Commons Attribution 4.0 International license.

The data stored in HVint2.0 contain annotation for a total of 918 PPIs (369 for HSV-1, 222 for HCMV, and 327 for EBV), including protein annotation, confidence scores, supporting evidence, and cross references to external databases. Also present in the database, is annotation of all 704 herpesvirus protein identifiers gathered during interactome reconstructions. The latter include proteins from the reference proteomes (as defined in the UniProt Proteomes database [13]) to which the final interactomes were mapped, as well as identifiers of proteins involved in the interaction from homologous species used for the homology mapping and reference proteome mapping. Similarly, the database holds annotation on associated data, such as taxonomic groups, PSI-MI (Proteomics Standards Initiative Molecular Interactions) ontologies (14), and primary citations (Fig. S1).

**FIG 2:**
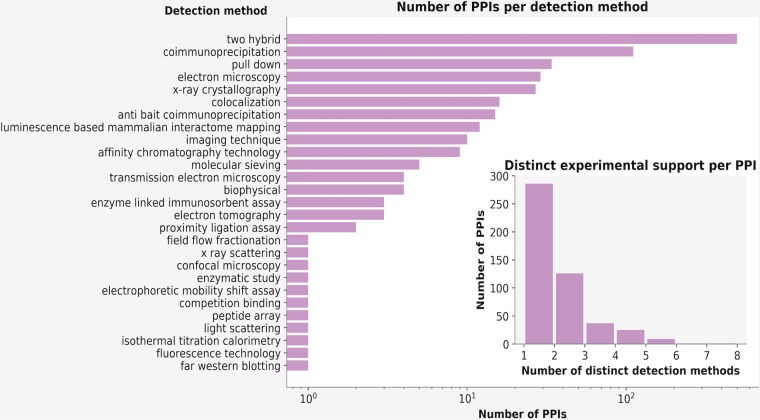
HVint2.0 content statistics. (A) Number of PPIs in HVint2.0 detected by each detection method (according to PSI-MI annotation). The main plot shows the number of PPIs in logarithmic scale. The counts for detection method categories “two hybrid” and “two hybrid array” have been joined in the same bar, as done similarly in the case of categories “coimmunoprecipitation” and “anti tag coimmunoprecipitation.” (B) Number of PPIs as a function of the number of different types of experimental detection methods supporting an interaction. Note that the graph reflects different types of evidence per PPI: i.e., if an interaction was detected by the same experimental method (for instance Y2H) more than once, this would still be counted only once.

Examining the contributions of different type of supporting evidence in HVint2.0 (Fig. 2A), we observe, as expected, that for the reconstructed interactomes, experimentally obtained supporting evidence is strongly populated with data resulting from yeast two-hybrid studies (Fig. 2A; see Fig. S3 in the supplemental material). However, nearly half of the PPIs in HVint2.0 are supported by data obtained from at least two different types of experimental methods (Fig. 2B). To test the ability and reliability of our PPI prediction approach in highlighting potentially new PPIs (yet to be experimentally detected), we conducted experimental testing on a subset of our interactions. The encouraging results of this study are discussed in detail in a related article (11).

10.1128/mSystems.00295-19.4 FIG S3 Number of interactions per primary citation. Here a large-scale study was defined as providing more than 50 PPIs. The graph shows that in the case of the reconstructed interactomes, only Y2H experiments provide that amount of interaction (corresponding to the four largest bars [6, 8, 68, 69]). Download FIG S3, TIF file, 1.1 MB. Copyright © 2019 Hernández Durán et al. 2019 Hernández Durán et al. This content is distributed under the terms of the Creative Commons Attribution 4.0 International license.

### Herpesvirus species-specific interaction networks.

**FIG 3:**
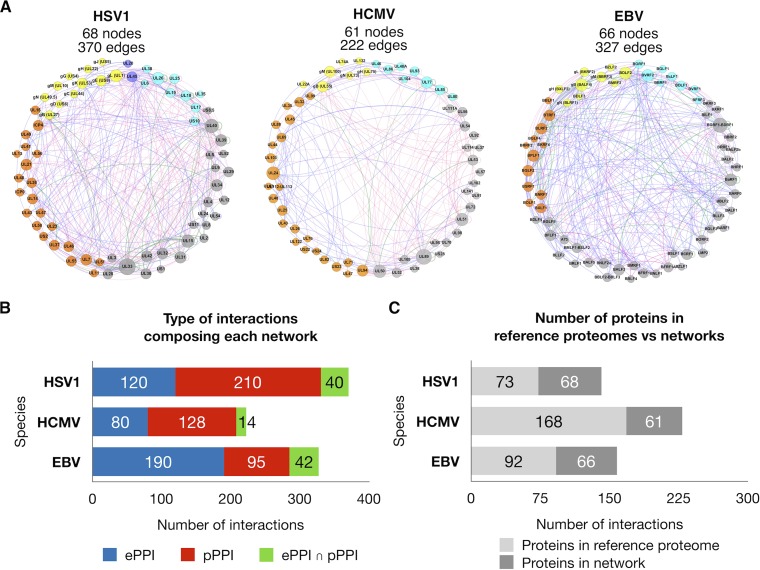
Herpesvirus species-specific interaction networks. (A) Reconstructed PPI interaction networks of HSV-1, HCMV, and EBV. Nodes (circles) are color coded as follows: cyan for capsid and capsid-associated proteins, orange for tegument, blue for nonglycosylated envelope proteins, yellow for envelope glycoproteins, and gray for nodes representing proteins not present in the mature virions. Node size is scaled between minimum and maximum within each network. Node labels contain protein open reading frame identifiers and popular glycoproteins in addition to their frequently used alternative names. Edges (links) are color coded according to the type supporting evidence for each interaction: red for computationally predicted PPIs (pPPIs), blue for experimentally supported PPIs (ePPIs), and green for interactions that have been both experimentally supported and computationally predicted (ePPIs ∩ pPPIs). The thickness of the edges is proportional to the confidence score of the interaction. (B) Bar charts for each species illustrating the number of PPIs as a function of their type of supporting evidence: i.e., red for pPPIs, blue for ePPIs, and green for ePPIs ∩ pPPIs. (C) Bar charts illustrating the size of the reference proteome (light gray) for each species (i.e., 73, 168, and 92 proteins for HSV-1 strain 17, HCMV Merlin, and Epstein-Barr B95-8, respectively) and the number of proteins from the reference proteome present in the network (dark gray).

The PPI network for each of the target species generated from HVint2.0 (Table 1 and Fig. 3A) contains a similar total number of nodes (proteins), as well as a similar number of nodes representing core (family-wise conserved) and noncore (sublineage-specific) proteins (40, 38, and 39 core protein nodes and 28, 23, and 27 noncore protein nodes in the HSV-1, HCMV, and EBV networks, respectively). The best-represented proteome is that of HSV-1, with ∼93% coverage, followed by those of EBV and HCMV, with ∼72% and ∼36% coverage, respectively. Analogous rankings are obtained if one considers core and noncore protein node fractions separately (Table 1).

**TABLE 1:** Reconstructed PPI networks in HSV-1, HCMV, and EBV

Species	No. of unique:		
	Pieces of evidence	PPIs	Protein sequences
HSV-1	644	370	68 (40 core, 28 noncore)
HCMV	393	222	61 (38 core, 23 noncore)
EBV	703	327	66 (39 core, 27 noncore)

### Inference of a herpesviral central interactome.

One of the features characterizing herpesvirus genomes across the *Alpha*-, *Beta*-, and *Gammaherpesvirinae* subfamilies, is the presence of a set of conserved proteins, referred to as “core” proteins. Following the consensus observed among available literature, we defined a list of 40 core proteins (see Table S1 and Table S2 in the supplemental material) (15, 16). Not surprisingly, these proteins are predominantly annotated with functional roles at early stages of the lytic life cycle, such as genome replication, packaging, capsid formation, and nuclear egress. However, they also include a few other components of the tegument and envelope, some of which are known to be responsible for processes that are imperative for an efficient viral production, such as glycoprotein B (gB) and the complex gH/gL, which are strictly required for entry into the host cell (17).

10.1128/mSystems.00295-19.6 TABLE S1 List of core herpesvirus proteins used in this study. Proteins in the same row are homologues. Download Table S1, XLSX file, 0.01 MB. Copyright © 2019 Hernández Durán et al. 2019 Hernández Durán et al. This content is distributed under the terms of the Creative Commons Attribution 4.0 International license.

10.1128/mSystems.00295-19.7 TABLE S2 Functional annotation for proteins in the central interactome. Data were retrieved from the HVint2.0 database. Download Table S2, XLSX file, 0.01 MB. Copyright © 2019 Hernández Durán et al. 2019 Hernández Durán et al. This content is distributed under the terms of the Creative Commons Attribution 4.0 International license.

**FIG 4:**
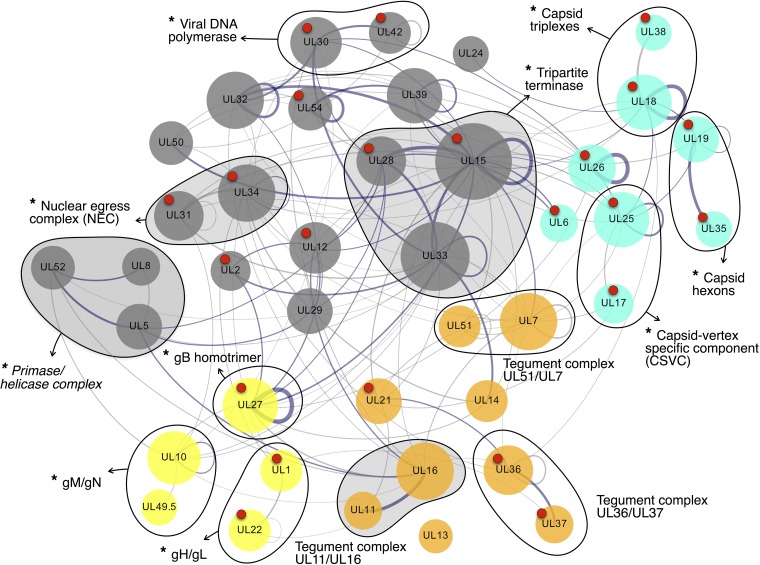
Inferred herpesviral central interactome. The network was obtained by compiling PPIs taking place among core proteins (Table S2) in at least two of the reconstructed networks for HSV-1, HCMV, and EBV. Edge thickness indicates PPI confidence scores, which were calculated as the sum of the scores for the interaction in each species it was found over the maximum number of species where the interaction could exist (i.e., 3). Nodes are color coded as follows: cyan for capsid and capsid-associated proteins, orange for tegument, yellow for envelope glycoproteins, and gray for proteins not present in the mature virion. Node labels correspond to HSV-1 open reading frame nomenclature. Node size is proportional to the number of interactions of each protein in the central interactome network. Red dots on top of nodes indicate the availability of structural data (full or partial reconstructions [Table S3]) for the corresponding node. Nodes circled together by a solid black line indicate the 13 family-wise conserved complexes represented by the interactome. Asterisks at the beginning of complex labels indicate obligate complexes (i.e., complexes for which its stable assembly is required for the proteins involved be functional). Complexes also detected in at least two species in reference 8 are indicated with shaded circles. Correspondences between ORF nomenclature, UniProtKB identifiers, and functionally informative tags for each protein can be found in Table S1 and Table S2.

All three species-specific networks reconstructed above contain nearly the full subset of 40 core proteins defined in this study (Table 2). The intersection across all the three subsets is of 37 (out of 40) proteins (nodes) and 56 interactions. However, to infer a potential herpesviral central interactome, here we considered PPIs occurring in at least two out of the three subsets (i.e., instead of the intersection of all three). The resulting network contained the full set of 40 core proteins and a total of 134 nonredundant PPIs (Fig. 4). Out of these 134 PPIs, 56 (∼42%) had experimental support (ePPIs or ePPIs ∩ pPPIs) in all three species and 89 (∼66%) in at least one species. Additionally, 45 PPIs (∼34%) had only been computationally predicted in all three (pPPIs only). Importantly, by taking into account PPIs present in at least two species but not requiring them to be in all three, the central interactome was able to overcome the major limitation of our PPI prediction strategy (i.e., the dependence on sequence similarity between proteins to infer their evolutionary conservation). Consequently, our reconstructed central interactome is able to suggest interactions that could not be predicted using a sequence homology-based approach. One example of this is the interaction for the heterodimer gH/gL in HCMV, which was missing when we originally (in 2016) collated the input data set to HVint2.0. Because gL orthologues are positional and functional homologues but lack detectable sequence similarity (18), they could not be predicted using a sequence similarity-based strategy. However, in 2017, the crystal structure of gH/gL in HCMV was solved (PDB ID 5VOD) (19), confirming our prediction.

**TABLE 2:** Species-specific core protein subnetworks

Species	No. of:	
	Nodes	Edges
HSV-1	40	192
HCMV	38	139
EBV	39	152

10.1128/mSystems.00295-19.8 TABLE S3 PDB entries associated with proteins and protein complexes appearing in the reconstructed *Herpesviridae* central interactome (Fig. 4). These entries can contain structural data for the full complex or only part of it (e.g., domains). Data obtained for homologous species are grouped in the same row using the HSV-1 nomenclature. Download Table S3, DOCX file, 0.02 MB. Copyright © 2019 Hernández Durán et al. 2019 Hernández Durán et al. This content is distributed under the terms of the Creative Commons Attribution 4.0 International license.

The generated central interactome contains 24 (60%) proteins that have some structural support (see Table S3 in the supplemental material). Furthermore, it is able to represent a total of 13 protein complexes widely accepted to be conserved across the *Herpesviridae* family, adding biological consistency to our results.

### Functional annotation of the complexes in the herpesviral central interactome.

**FIG 5:**
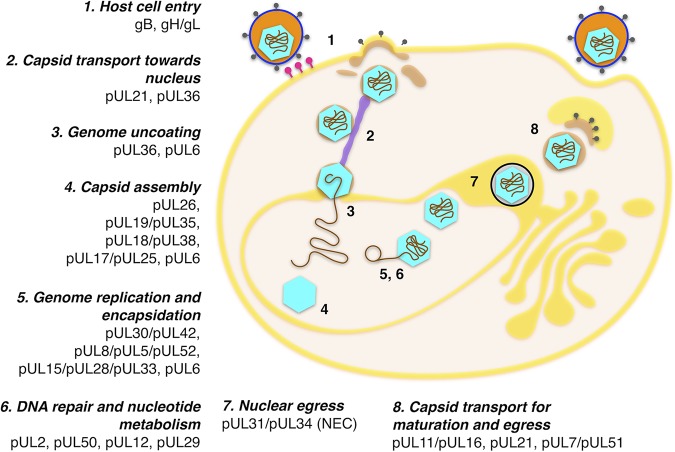
Central interactome complexes in the herpesvirus life cycle. The sequential steps in a herpesvirus lytic life cycle are indicated with numbers. The complexes present in the reconstructed central interactome participating in each of the stages are also annotated. Upon viral entry mediated by virus envelope-cell membrane fusion (step 1), the tegument is delivered into the host cell and capsids are transported to the nucleus (step 2). Docking to the nucleopores triggers genome uncoating (i.e., delivery to the nucleus) (step 3). In a lytic replication, a procapsid is formed (step 4), and genome replication, capsid maturation, and genome encapsidation occur concomitantly (steps 5 and 6). Capsids egress from by budding at the nuclear membranes (step 7). The bulk of the tegument is acquired in the cytoplasm, primarily by fusion with *trans*-Golgi network-derived vesicles (step 8). Fusion of these vesicles with the plasma membrane upon virion maturation releases the virions to the extracellular space (step 8). Correspondences between ORF nomenclature, UniProtKB identifiers, and functionally informative tags for each protein can be found in Table S1 and Table S2.

The 13 complexes composing the central interactome (Fig. 4 and 5) are predominantly involved in processes such as DNA replication and insertion of the genome into the viral capsid, formation of the latter, invasion of new host cells, as well as key aspects of virion structure and morphogenesis. Complexes involved in DNA replication and packages include the tripartite complexes helicase/primase (complex 1) and tripartite terminase (complex 2). These are responsible, respectively, for double-helix unwinding and genome packaging into new viral capsids, prior to and after genome replication by the DNA polymerase complex (complex 3), which is also present in the central interactome (20–22). Capsid complexes include interactions among hexon subunits (complex 4), heterotrimeric triplexes (complex 5) surrounding capsid pentons, and the capsid-vertex-specific component (CSVC) (complex 6) (23). Also present is the nuclear egress complex (NEC) (complex 7), which is required and sufficient to facilitate budding of the nuclear capsids at the inner nuclear membrane for primary envelopment (24). Tegument complexes known to participate at different stages of secondary envelopment include the UL36 and UL37 complex (UL36-UL37; complex 8), UL11-UL16 (complex 9), or UL51-UL7 (complex 10) (25–28). Finally, glycoprotein complexes such as gM/gN (complex 11) (29), and the abovementioned gH/gL (complex 12), suggested to act as the activity regulator to the trimeric fusogen gB (complex 13) (17), are also present. One complex not found in the central interactome, which surprised us initially, is the ribonucleotide reductase (RNR) complex, formed by homologues of HSV-1 proteins UL39 and UL40 (R1 and R2 subunits, respectively) (30). RNR is a holoenzyme that catalyzes the formation of deoxyribonucleotides required for DNA synthesis, and it is essential for viral growth in both *Alpha*- and *Gammaherpesvirinae* subfamilies. However, the reason this enzyme is missing from the central interactome is that an open reading frame (ORF) for the R2 subunit is missing in genomes of the *Betaherpesvirinae* subfamily (31). Consequently, the node corresponding to the R2 subunit does not appear in our network, and therefore the complex cannot be inferred.

Previously, published data on PPIs among core proteins in herpesviruses (8) defined a central interactome by including all PPIs taking place among core proteins in one or more of five analyzed herpesvirus species. To compare our data set to theirs, we first applied the same constraints as ours to their data set: i.e., we only considered interactions that were detected in at least two species among HSV-1, murine cytomegalovirus (MCMV), and EBV. (Note that the experiments in reference 8 were conducted 10 years ago, and there the authors used MCMV as a *Betaherpesvirinae* subfamily representative instead of HCMV.) We found that the coverage of the central interactome reconstructed in our study is in fact larger, both in terms of total number of PPIs (134 PPIs versus 17 PPIs in reference 8) and the number of conserved protein complexes.

### HVint2.0 web interface.

**FIG 6:**
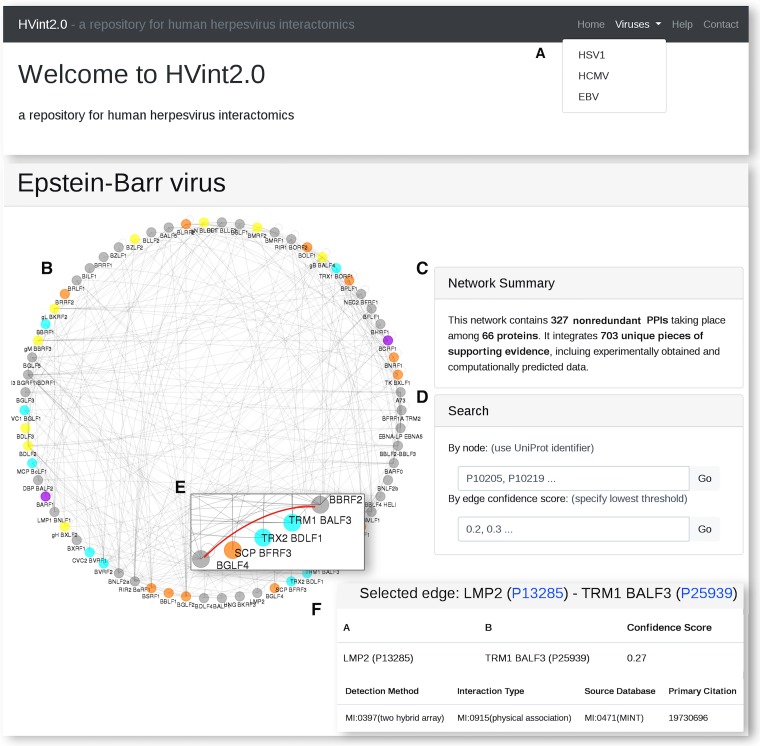
HVint2.0 web interface. (A) Each interactome can be accessed from the top dropdown menu. (B and C) For each interactome, the website provides both a graphical representation of the network and a brief summary. (D) Search tools to subset the full network based on node identifiers or edge confidence threshold. (E) Alternatively, specific nodes and edges of interest can be selected from the graphical display. (F) For each selection, the corresponding subnetwork is rendered and its associated data provided in tabular format.

The HVint2.0 web interface (http://topf-group.ismb.lon.ac.uk/hvint2) provides an intuitive user-friendly access to the reconstructed interactomes (Fig. 6). From the main page, the user can access the reconstructed interactomes via a dropdown menu (Fig. 6A). Each interactome page is designed to provide the user with both a graphical representation of the network data (Fig. 6B) and a brief summary of the inspected interactome (Fig. 6C), as well as tools for further exploration, such as search boxes for both nodes and confidence score thresholds (Fig. 6D). However, one can also interactively explore the network by clicking nodes and edges in the graphical display window (Fig. 6E). Upon each query, the page is updated to provide both a graphical representation of the subnetwork associated with the query and further annotation data on these interactions in tabular format, including supporting evidence (Fig. 6F).

## DISCUSSION

### Study rationale.

The motivation behind our study was 2-fold. First, we wanted to introduce the extended HVint2.0 database, a centralized resource of curated PPI data across the *Herpesviridae* family. The database now includes data on archetypical species of all three phylogenetic herpesviral subfamilies: HSV-1 for the *Alpha*-, HCMV for the *Beta*-, and EBV for the *Gammaherpesvirinae*. To populate HVint2.0, we compiled new system-level PPI data sets for each of the three selected species using our recently updated network reconstruction framework (11).

Second, we wanted to use the compiled PPI data to extract new biological knowledge. Specifically, taking advantage of the fact that the collected PPI data span the *Herpesviridae* phylogeny, we sought to infer the family-wise conserved fraction of these networks, i.e., a “central” human herpesvirus intraviral interactome. PPIs are now consolidated as major driving forces behind observed phenotypes. Identification of the features that distinguish two or more biological networks can help explain species-specific pathogenic strategies and phenotypes. Equally important is to characterize the common network features across different species networks, as this fraction would presumably contain the scaffolding components that allow establishing an infection in different host systems.

### Network coverage.

The total number of nodes, as well as core and noncore proteins represented in the three reconstructed networks, are comparable. However, they translate into different levels of proteome coverage for each network. The most comprehensively represented proteome is that of HSV-1, followed by EBV and HCMV. The same still holds for the fractions of the intraviral interactome formed by core and noncore proteins, for each species (which correspond, respectively, to the family-wise and sublineage-specific fractions of the proteomes). In the case of HSV-1 and EBV, a significant proportion of their sublineage-specific proteomes (∼85% and ∼52%, respectively) are present in the assembled networks. Instead, for HCMV, only around 18% of it is present. There are two main reasons for that. One is that the HCMV proteome is significantly larger (168 canonical protein sequences in the UniProt reference proteome) than those of other herpesviruses, including HSV-1 and EBV (with 73 and 92 proteins in their reference proteomes, respectively). The other reason is a remarkable underrepresentation of experimentally supported data for members of the *Betaherpesvirinae* subfamily in the input data set (Fig. 3B), which could derive, for instance, from experimental limitations in efficient culturing of species of the *Betaherpesvirinae* subfamily. Therefore, our results expose the areas in these herpesviral interactomes that are least represented by currently available data and would benefit most from further investigation.

### Reconstruction of a central interactome and functional annotation.

To reconstruct our herpesviral central interactome, we used a cross-species network comparison and integrated all the interactions among core proteins. We have compared the central interactome with that of previously published data from reference 8. In the latter study, the authors conducted what constitutes, to the best of our knowledge, the largest experimentally based study on herpesviral networks to date and which has been a valuable input toward our network reconstructions. After applying the same constraints to their initial data set as the ones we used in our own reconstruction (i.e., PPIs among core proteins taking place in at least two of the three studied species), we found that the central interactome reconstructed in our study contains a larger number of PPIs, including a larger number of known conserved herpesviral protein complexes. These results are encouraging, indicating that our strategy of integrating experimentally and computationally generated data increases the coverage of our interactome in a biologically consistent and informative manner.

The choice of including PPIs present in only two of the three networks instead of all of them partially compensates for the limitations derived from a sequence-based PPI prediction method for orthologous proteins with very low or nonexistent sequence similarity, such as in the case of positional homologues like gL (18). Experimental data supporting the well-known interaction between gL and gH in HCMV specifically were missing in our input data set. Because of the lack of sequence similarity among gL homologues, the interaction between gL and gH could not be mapped from other species using a sequence-based approach. Yet, the reconstructed central interactome was able to highlight this interaction as family-wise conserved, which was later validated experimentally (19).

In total, the reconstructed central interactome contains representations of 13 protein complexes known to be conserved across the family. Unsurprisingly, these complexes are involved in essential functions for a productive infection, such as capsid formation, genome replication and encapsidation, virion egress, and viral entry. Together, it is easy to envision how these complexes could suffice as an essential toolkit for the creation of a minimal yet productive viral particle. This would consist of a basic self-assembling capsid shell containing a copy of the viral genome, which would then be able to egress the infected cell, circumvent the immune surveillance in the extracellular media, and finally deliver the viral genome into a new host cell. In contrast, the species-specific fractions from each of the reconstructed networks show well-defined functional differences from the central interactome (see Table S4 and Fig. S4 in the supplemental material). Comparatively, these species-specific fractions are enriched in proteins involved in pathways that are likely to have evolved, to a larger degree, to increase the efficiency of the infection in a host-specific manner. Examples of such processes include immune modulation, cell tropism, virulence, or gene expression regulation.

10.1128/mSystems.00295-19.5 FIG S4 Inferred herpesviral central interactome and species-specific subnetwork. The inferred herpesviral central interactome is shown at the center, flanked by the subsets of species-specific interactions for each of the three species from the reconstructed interactomes. Gray edges and nodes indicate PPIs and proteins found in the central interactome. These are referred to using HSV-1 open reading frame nomenclature. Red, blue, and green edges and nodes indicate PPIs and proteins from the species-specific subnetworks in HSV-1, HCMV, and EBV, respectively. Edge thickness reflects confidence scores. For central interactome PPIs, this score was calculated as the sum of the scores for the interaction in each species it was found over the maximum number of species where the interaction could exist (i.e., 3). In species-specific subnetworks, this is calculated using the scoring function integrated in our network reconstruction framework. Node size is proportional to the number of interactions of each protein. Download FIG S4, TIF file, 1.4 MB. Copyright © 2019 Hernández Durán et al. 2019 Hernández Durán et al. This content is distributed under the terms of the Creative Commons Attribution 4.0 International license.

10.1128/mSystems.00295-19.9 TABLE S4 Functional annotation for proteins in all three species-specific network fractions. Data were retrieved from the HVint2.0 database. Download Table S4, DOCX file, 0.05 MB. Copyright © 2019 Hernández Durán et al. 2019 Hernández Durán et al. This content is distributed under the terms of the Creative Commons Attribution 4.0 International license.

The concept of a minimal biological system has been explored for a long time with the goal of unraveling the fundamental basis of life and the function of individual genes (32, 33). In this context, different strategies have been explored, including, but not limited to, minimal cells (32), *in silico* cell models (34), genomes (35), and minimal sets of genes (36). Within the virosphere, the initial interest largely focused on synthetically recreating minimal self-assembling capsid-like structures, mainly due to the potential as nanocarriers for drug delivery (37, 38). In 2016, Bale et al. (39) reported the creation of stable and engineerable icosahedral protein shells with enough internal volume to enclose biological macromolecules, such as their own encoding nucleic acids (39–41). Subsequent experiments also demonstrated the capacity of such RNA-protein complexes to evolve improving their fitness, providing new hypothetic evolutionary scenarios that could have given rise to ancestral viral particles (41). Contemporary viruses exhibit a much larger and complex array of functionalities that have allowed their adaptation to different environments and hosts, and at present, ample room remains to be explored to understand such evolutionary paths leading to such complexity. In a similar fashion to the case of cellular organisms, a first step to decode the currently observed viral complexity, of which herpesviruses are outstanding examples, is to identify those elements that are part of the essential scaffold (core genes and proteins), which viral evolution built upon over time.

In this study, we propose a scaffolding central interactome for the *Herpesviridae* family, one of the most structurally and genetically complex family of viruses (1). These data contribute to characterizing the evolutionary history of these human pathogens. Combining the central interactome with the species-specific networks, our data will assist in creating comprehensive system-level models of the functional architecture of this viral lineage. As computational models inspired on minimal cellular systems have already been demonstrated (42, 43), in this case, these models can bring new insights into pathogenic behaviors and phenotypical outcomes, guide experimental analysis, and potentially become important for designing and testing new molecules for industrial and clinical application.

### Species-specific networks.

It is beyond the scope of this article to delve into the numerous species-specific features that characterize different herpesviruses. However, in the paragraphs below we elaborate on some of the examples that our species-specific network reconstruction highlights and encourage the reader to explore the vast and interesting literature available on the subject.

### (i) Initiation of lytic replication.

We observe for instance, proteins involved in species-specific pathways that launch the gene expression cascades leading to lytic replication. In HSV-1, for instance, protein pUL48 (also called VP16) acts as a transcriptional activator, stimulating the expression of immediate-early (IE) genes (44), such as protein pUS1 or ICP0 (also present in the species-specific subnetwork).

In HCMV, proteins pUL122, pUL37, pUL38, and pUL112-113 constitute replication auxiliary factors only conserved in the *Betaherpesvirinae* subfamily. pUL122-123 is the precursor for the major immediate-early (MIE) genes, IE1 and IE2 (45). Proteins such as pUL37 and pUL38 have been suggested to contribute to viral replication by promoting the maintenance of essential cellular functions (46). The pUL112-113 genes encode four different phosphoproteins obtained through alternative splicing and can enhance DNA replication both by binding the DNA and promoting the transcriptional activation of IE genes, as well as by direct interaction with the latter products and the DNA polymerase (47).

The EBV species-specific subnetwork contains, in turn, the two IE transcriptional activators encoded by this virus, BRLF1 and BZLF1. Expression of either of these proteins has been shown to be sufficient to induce lytic replication from latency.

It is interesting to note that although the proteins referred above are encoded in a species- or sublineage-specific manner, some of the mechanisms they use to exert their functions are shared across species. For instance, the transactivators encoded in the three subfamily representatives (i.e., VP16 in HSV-1, IE2 in HCMV, and BRLF1/BZLF1 in EBV) all bind CREB-binding protein (CBP), to enhance transcription through histone acetylation (48).

### (ii) Tropism.

In HSV-1, pUS6 (gD) is the HSV-1-specific receptor-binding protein that triggers the signal for membrane fusion to occur. pUL44 (gC) is an *Alphaherpesvirinae*-specific protein known for its role in host cell viral entry, where it promotes initial attachment of the virion to the cell surface by binding heparan sulfate proteoglycans, but it also exerts a function as a virulence factor by inhibiting classical and adaptive host immune responses (49). In EBV, we find BZLF2, also known as gp42, which controls cell tropism. The complex between gH/gL and gp42 enables infection of B cells; however, the gp42 N terminal inhibits entry in epithelial cells (50). Although the complexes driving HCMV tropism (i.e., the pentamer gH/gL/pUL128-131 [which includes UL128 through UL131] and the trimer gH/gL/gO) had been functionally characterized previously, conclusive evidence of the physical binary interactions taking place among the respective components was not available at the time of our data collection. The crystal structure of gH/gL/pUL128-131 was only solved in 2017 (PDB ID 5VOD) (19), and structural details for gH/gL/gO are, at this time, limited to low-resolution data (51).

### (iii) Interference with host defense mechanisms.

Other proteins are involved in counteracting the host immune defense mechanisms. The pUS4 (gG) and pUS5 (gJ) genes in HSV-1 encode glycoproteins acting as immunomodulators. gG is a viral chemokine-binding protein (vCKBP), binding human chemokines expressed at the sides of viral replication and spread with high affinity (52), which translates into increased cell migration to these sites. The advantage that this mechanism confers to the virus is still unknown, but it has been suggested that it could help recruiting new infection targets, facilitating viral load and spread (52). Although the specific mechanisms by which gJ exerts its function are not fully understood, it is known to be sufficient to prevent apoptosis induced by several stimuli, including UV, fas, or cytotoxic T cells (53). The HCMV protein pUL25 antagonizes interferon responses, for instance by antagonizing the action of interferon-stimulated gene 15 protein (ISG15), which prevents the proteasomal degradation of other viral counterparts, such as pUL26 (54). In EBV, proteins BHRF1 and BALF1 are homologues of the mammalian cell death inhibitor BCL-2 proteins and participate in regulating apoptosis in infected cells (55).

### (iv) Other.

We also find proteins like pUL23 in HSV-1 and BXLF1 in the EBV subnetworks, which encode the viral thymidine kinase. Thymidine kinase is commonly known for its role as an antiviral drug target. During infection, it participates in the metabolism of nucleic acids, catalyzing the conversion of thymidine (Thd) into dTDP (TDP) (56). To date, herpesvirus thymidine kinase ORFs have only been detected in the *Alpha*- and *Gammaherpesvirinae* subfamilies but could not be found in most *Betaherpesvirinae* genomes, including HCMV (57). In HCMV, the pUL32 gene encodes the basic phosphoprotein (BPP, or pp150), homologues of which are only found within the *Betaherpesvirinae* subfamily but not in the *Alpha*- or *Gammaherpesvirinae* lineages. pUL32 is closely associated with the capsid and constitutes a major constituent of the HCMV virion. Although not essential, this protein has been show to play a very important role in the correct morphogenesis of newly formed virions (58).

### HVint2.0 usage.

This study has presented comprehensive compilations of curated system-level PPI data for three representative species across the *Herpesviridae* phylogeny. Full interactome coverage is, at present, still a distant target for individual techniques, and repositories and integrative pipelines like the one underlying the HVint2.0 data sets are needed to alleviate such shortcomings. The integration of computationally predicted PPIs does not only increase the coverage of the resulting networks. These data can also assist in more confidently identifying nonspecific interactions and false positives among experimentally generated data, such as those obtained from immunoprecipitation-mass spectrometry (IP-MS) studies (59). Our PPI network reconstruction framework presents limitations that are worth bearing in mind, such as the dependency on detectable sequence similarity between homologous proteins. However, the output of the current study already provides useful tools and data to help answering questions on the evolutionary dynamics of this important group of eukaryotic viruses and human pathogens. The data generated by our analysis could be the basis of several projects across a number of disciplines, including, but not limited to, PPI discovery, evolutionary dynamics, and structural modeling of the involved networks. Further, the availability of three-dimensional structural data for over half of the components in the central interactome encourages prospective endeavors to structurally model the full network. This will add another level of understanding of the dynamics and constraints that govern proteins’ connectivity.

### Summary.

In the protocol described here, we strived to maximize not only the coverage of our reconstructed networks but also the degree of automation of the pipeline so that it is easily reproducible and generalizable to other species. This is the main reason why we minimized the use of manual literature curation in our protocol. Another reason is that, despite the advantages that such curation may bring (such as a larger degree of PPI coverage), this could also entail major drawbacks, especially regarding data quality validation and frequency of new database releases. Therefore, in our pipeline we used data repositories with dedicated curation teams that follow the curation rules established by the IMEx consortium (https://www.imexconsortium.org/) or that directly pull their data from such curated repositories. Taking advantage of the fact that our pipeline has been designed to be easily reproducible in other species (within the *Herpesviridae* and from other viral families), we plan to regularly release database updates, keeping up to date with new updates of the input PPI databases.

## MATERIALS AND METHODS

### Database design.

We shall first emphasize the distinction between the back-end HVint2.0 relational database (see Fig. S1 and Text S1 in the supplemental material) and the HVint2.0 web interface. The HVint2.0 relational database contains all the data collated during the study. These include a range of diverse annotation data, in addition to the finally reconstructed intraviral interactomes, such as for instance, functional annotations for proteins, primary citation details (like authors and journals), or protein sequence annotations, among others. While these additional data are certainly relevant, and we encourage the reader to explore them, navigating through these details can become cumbersome—especially at the early stage of network exploration. Our goal when creating the HVint2.0 web interface was precisely to ease the exploration of synthetic yet comprehensive representations of the interactomics data in our database, These representations hold all the key information to understand the evidence behind each interaction and trace its original source.

The HVint2.0 relational database was created using the MySQL server (version 5.1.73) as a relational database management system, within a Linux platform. The data are distributed across a total of seven tables. Six tables as named represent “protein,” “PPI,” “taxon,” “evidence,” “citation,” and “psimi_ontology” entities (Text S1 and Fig. S1). The remaining table allows referencing many-to-many relationships between “evidence” lines and the “psimi_ontology” table. Protein annotation, taxonomic data, and citation details were collated from the UniProtKB (13), NCBI Taxonomy (https://www.ncbi.nlm.nih.gov/taxonomy), and PubMed databases, respectively. Cross-references to the input databases are annotated following the PSI-MI format.

Although the PSI-MI format allows to allows annotation of each binary interaction with multiple pieces of annotation, for the implementation of our pipeline, the following are strictly necessary: identifiers for both of the proteins participating in the interaction, the PPI detection method (e.g., yeast two-hybrid [Y2H]), the type of PPI (e.g., colocalization), and the primary citation identifier (e.g., the PubMed identifier of the study reporting the interaction).

### Data collection, PPI prediction, data integration, and scoring of PPIs.

To reconstruct the network for each of the three target species of human herpesviruses (i.e., HSV-1, HCMV, and EBV, respectively), we used our recently developed automated pipeline for collecting PPI data in HSV-1 in an unbiased fashion (11) (Fig. S2). In short, input data were retrieved from five molecular interaction repositories—BioGRID (60), the Database of Interacting Proteins (DIP) (61), IntAct (62), Mentha (63), and VirHostNet 2.0 (64)—and two structural databases—the Protein Data Bank (PDB) (65) and the Electron Microscopy Data Bank (EMDB) (66). To minimize bias, filters to ensure the quality of the PPI data were only applied by us to PPIs obtained from the structural repositories. These databases are not dedicated specifically to molecular interaction data, and therefore, we inspected each entry individually and selected binary PPI only where the following criteria were met:

- Entries provided structural evidence of a physical interaction between protein chains and not other types of molecules: i.e., interactions involving nonpolypeptidic molecules, such as nucleic acids or small molecules, were disregarded.
- Entries had an associated PubMed ID: i.e., deposited structures without associated publication at the time of data collation were disregarded.
- The interacting chains belonged to one of the input species considered in our pipeline (i.e., human herpesviruses, as well as pseudorabies virus [PRV], MCMV, and murine gammaherpesvirus 68 [MHV68]).

In the case of PDB entries, we also looked for atomic contacts (as defined by default in UCSF Chimera [67] between protein chains belonging to the herpesvirus species considered in our study—i.e., all human herpesviruses, as well as pseudorabies virus, murine betaherpesvirus, and murine gammaherpesvirus). In the case of EMDB entries, we only considered those at near-atomic resolution at the time of data collation (i.e., a resolution of ≤5 Å).

For each of the target species, we used PPIs experimentally detected in orthologous species to predict new interactions in the target interactome. Mapping of binary interactions between two species relies on the existence of the corresponding homologous pairs of proteins in each of those species. Our homology mapping strategy combines information on the alignment coverage, sequence identity and similarity, and probabilistic scores to estimate the likelihood of a hit of being a true-positive homologue. The resulting predictions were then integrated (removing redundancy) with PPIs experimentally detected in the target species (11). It is worth noting that the input databases and the homology mapping sometimes involved protein isoforms, and in those cases, we mapped them to the canonical ORFs.

Each of the PPIs was scored using our recently implemented scoring function, which relies on MIscore (12), taking into account the heterogeneity in the evidence supporting an interaction, and an additional scaling factor to penalize for computational predictions (11). The latter includes information on the prediction method used (i.e., sequence-based orthology) and follows more closely the (nonlinear) mathematical description of the terms in the MIscore function.

The output data of all three networks are stored in HVint2.0 (http://topf-group.ismb.lon.ac.uk/hvint2). The entire pipeline, which is implemented as a series of python scripts using standard (default) libraries, is reproducible and can be generalized to include other species.

### Inference of a herpesviral central intraviral interactome.

For each of the networks, we extracted the subnetwork composed of core proteins. Here we define the core protein data set as a total of 40 proteins based on previously published literature (Table S1 and Table S2) (15, 16). A central intraviral interactome was next assembled by gathering all PPIs present in at least two of the three subnetworks; both experimentally supported and computationally predicted were equally considered. To assess the completeness and biological coherence of the reconstructed central interactome, we assessed the extent to which widely accepted conserved protein complexes in herpesviruses found in the literature were represented in the central interactome network.

### Construction of the HVint2.0 web interface.

The full pipeline is implemented as a series of python scripts using standard (default) libraries.

The HVint2.0 web interface was built using the open source library for web development Bootstrap (https://getbootstrap.com/), which provides an easy-to-implement framework for development of responsive databases. A static JSON-formatted representation of the network data is rendered using the JavaScript library vis.js (http://visjs.org/), designed to ensure correct rendering in several web browsers.

### Data availability.

The data sets produced in this study are available by clicking on the “Viruses” dropdown menu at http://topf-group.ismb.lon.ac.uk/hvint2.
