# Supplementary figures and images for: Conserved Central Intraviral Protein Interactome of the Herpesviridae Family
Source: mSystems. 2019 Oct 1;4(5):e00295-19. doi: 10.1128/mSystems.00295-19 (PMC6774017; doi:10.1128/mSystems.00295-19)

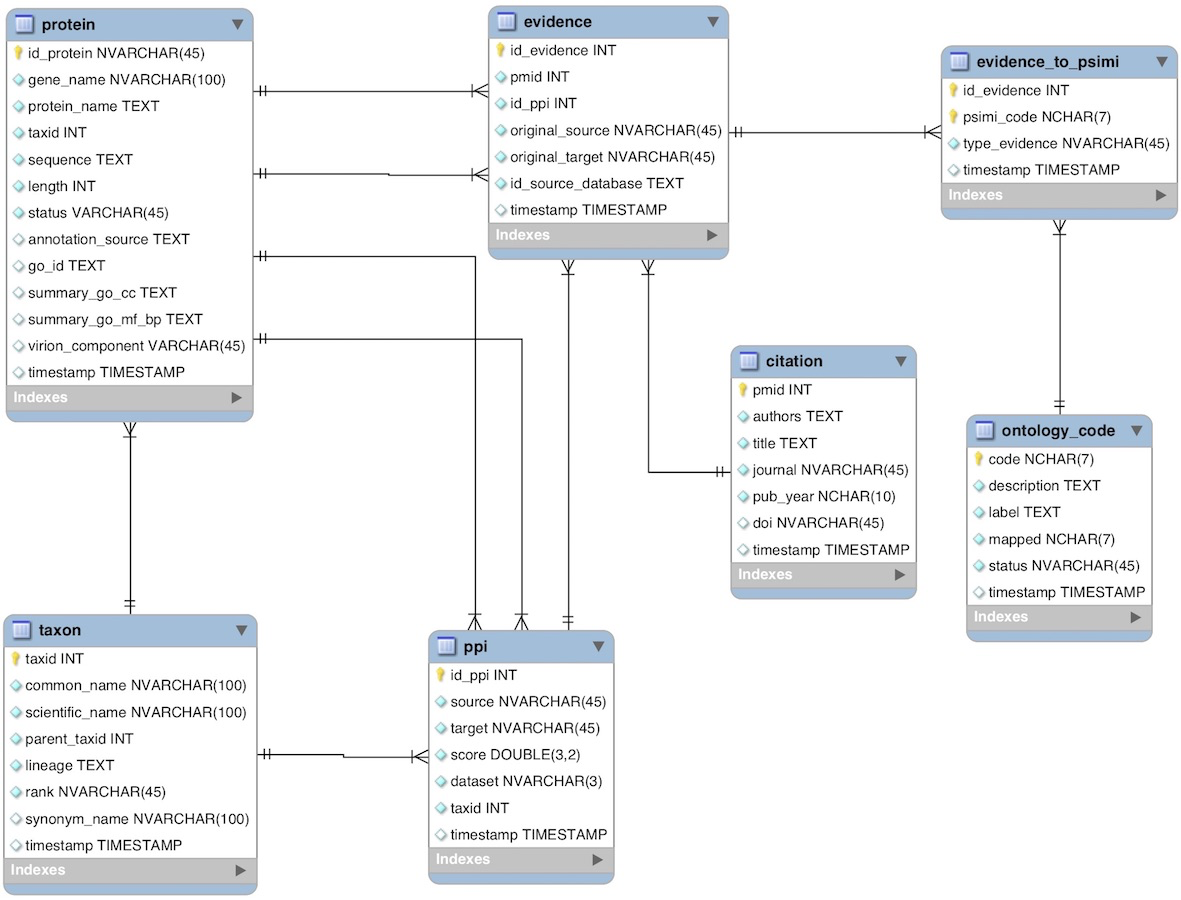

Supplement: FIG S1 [file mSystems.00295-19-sf001.tif]

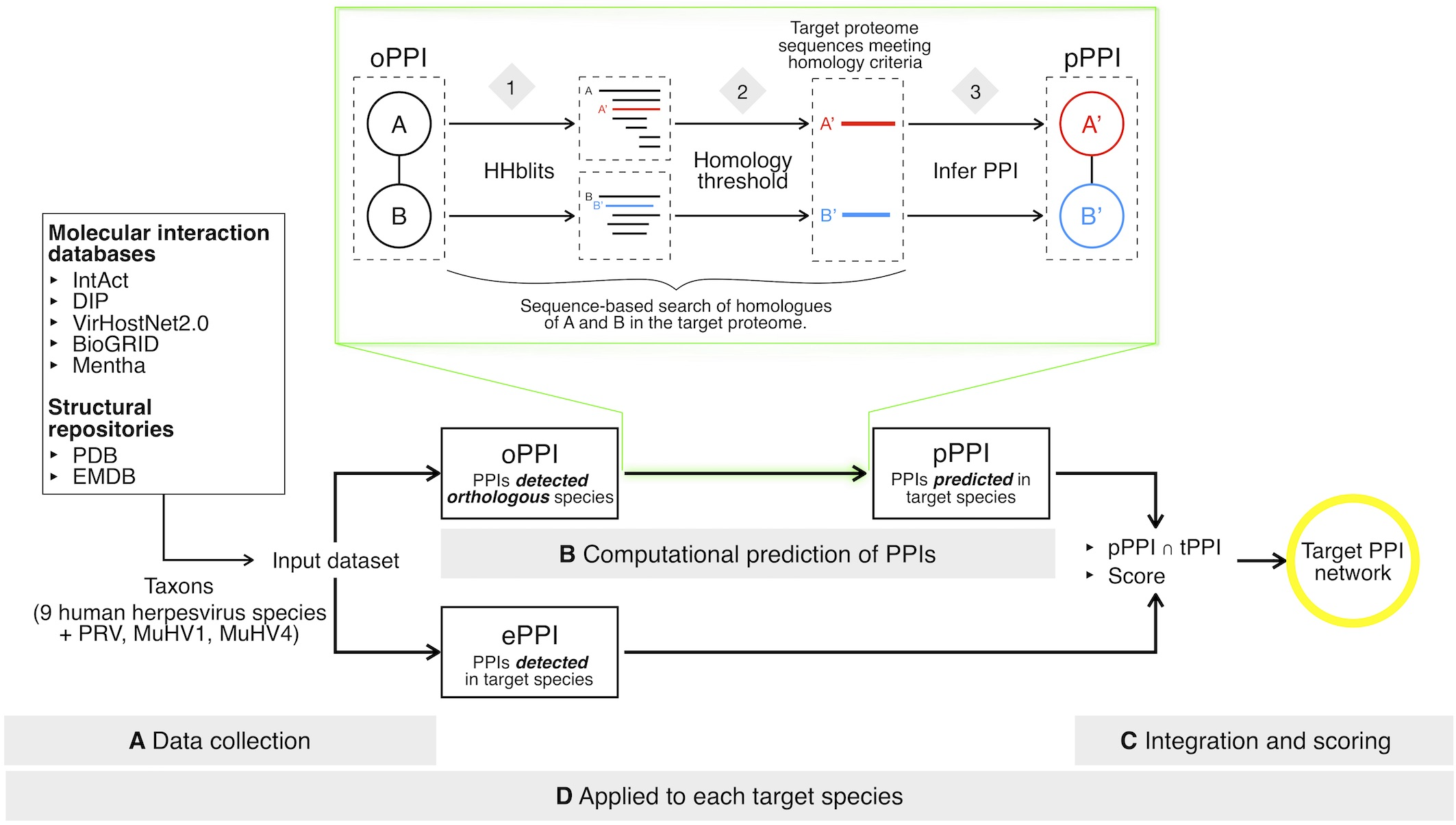

Supplement: FIG S2 [file mSystems.00295-19-sf002.tif]

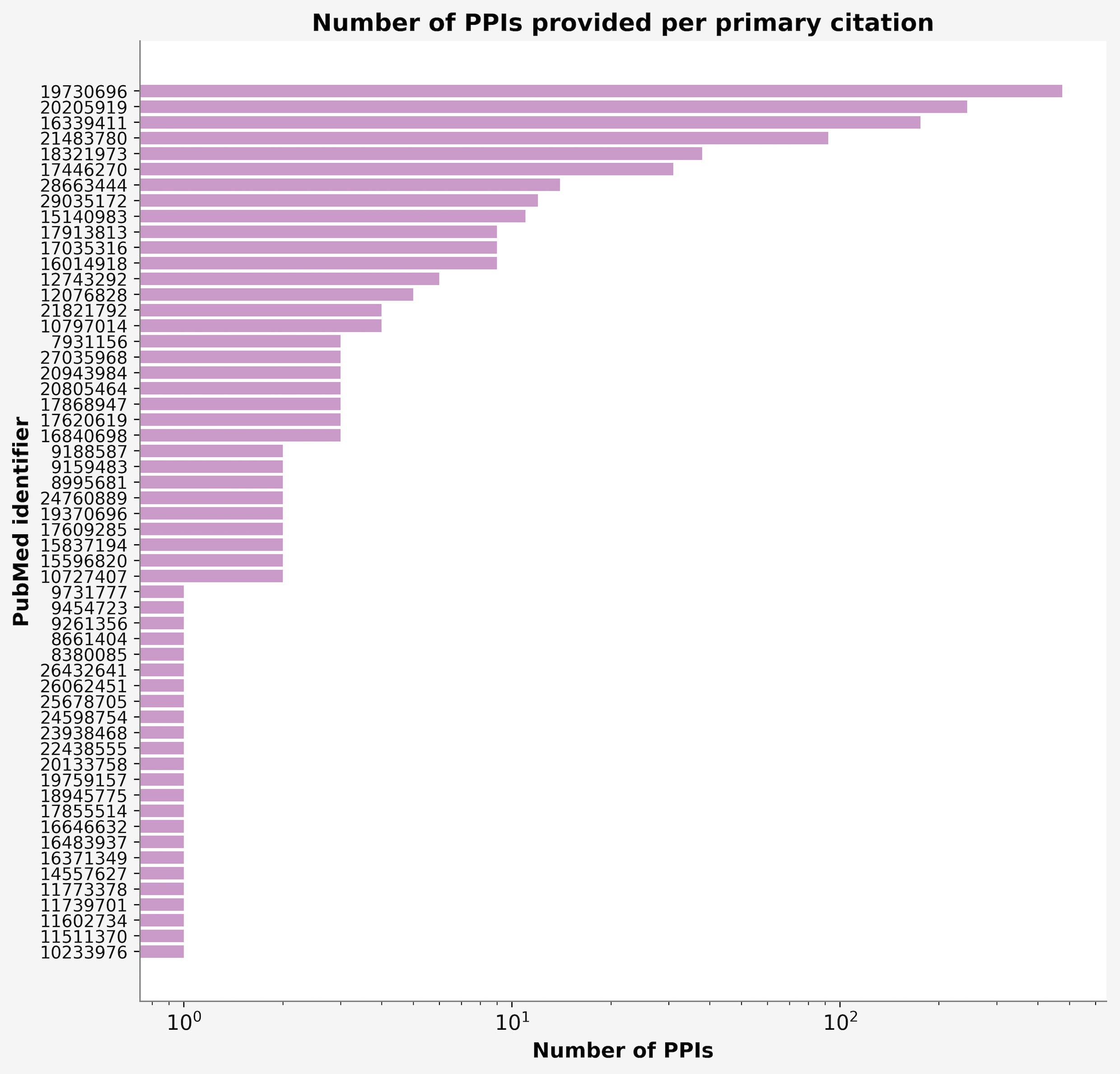

Supplement: FIG S3 [file mSystems.00295-19-sf003.tif]

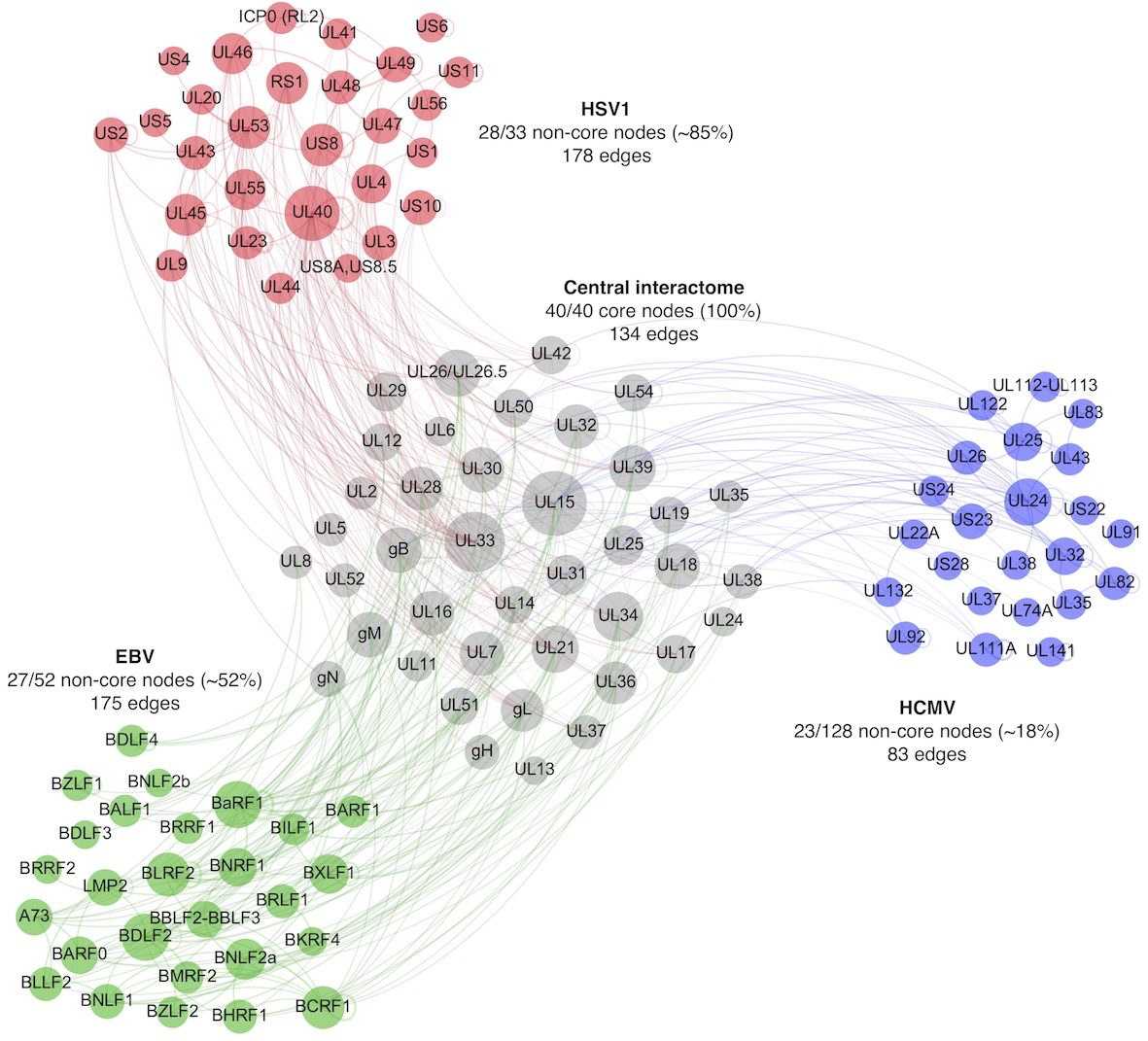

Supplement: FIG S4 [file mSystems.00295-19-sf004.tif]
