# Supplementary material for: Conserved Central Intraviral Protein Interactome of the Herpesviridae Family
Source: mSystems. 2019 Oct 1;4(5):e00295-19. doi: 10.1128/mSystems.00295-19 (PMC6774017; doi:10.1128/mSystems.00295-19)
Supplement: TEXT S1 [file mSystems.00295-19-s0001.docx]

**SUPPLEMENTARY TEXT**

**Text S1. Database structure and content**

The data in HVint2.0 are all organized around the *ppi* table. This central table contains records for each of the PPIs (918 in total) in the three reconstructed interactomes. Each interaction in the table is described with the protein identifiers (from reference proteomes) of source and target nodes, the confidence score of the interaction, the taxonomic identifier or the interaction, and the type of supporting evidence (dataset column) indicating whether this is experimentally obtained (E), computationally predicted (P), or the interaction holds both types of supporting evidence (E+P).

The database contains annotation data for a total of 704 proteins, including UniProtKB identifiers, protein and gene names, taxonomic identifiers, canonical protein sequences and their lengths, and the status of the protein entry in the UniProtKB database (i.e. whether it is reviewed, unreviewed, or obsolete). These 1589 include proteins from the three reference proteomes for which a network was reconstructed in this study (HSV1, HCMV, and EBV, obtained from UniProtKB Proteomes database (Consortium, 2017)), plus all other protein identifiers collected throughout the networks reconstruction process. This means that protein identifiers present in PPI supporting evidence prior homology or reference strain mapping using UniRef90, are also present in the table. Protein identifiers of source and target nodes in the reconstructed interactomes are additionally annotated with functional and localisation annotation, both in cellular components and in the virion particle, obtained from manual curation of Gene Ontology (GO) annotation records and additional relevant literature. Specifically, this extra annotation includes GO identifiers, references to the annotation sources (GO, literature PubMed identifiers, etc.). These 1589 proteins belong to a total of 27 different phylogenetic taxons, which are in turn annotated in the taxon table. This table describes each taxon with their taxonomic identifier, common and scientific names, taxonomic rank, and lineage, and includes as well the parent taxon taxonomic identifier for each taxon in the table.

The *psimi_ontology* table contains 1108 entries, corresponding to each term in the Molecular Interactions Controlled Vocabulary ontology (Kerrien *et al*, 2007). The table contains PSI-MI codes, descriptions and labels for each term. To represent the hierarchical organisation of the ontology the table also includes, for each term, the PSI-MI code of its parent term (except for the root term, indicated as ‘root’), and the depth level from the root term, i.e. the shortest path length from the node to the root. To keep track of potential updates in the PSI-MI codes, the table contains a status and mapped columns, which indicate whether an ontology term is active or obsolete, and which ontology code obsolete codes are mapped to if applicable.

The *evidence_to_psimi* table is an intermediary table to handle the many-to-many relationships between the *evidence* and the *psimi_ontology* tables, i.e. each evidence record can be associated to more than one ontology code and *vice versa* (5488 records in total). These intermediates tables are a requirement in relational databases for them to be able to handle many-to-many relationships. This table contains three columns, associating pairs of evidence id and psimi code, and annotating the pair by the type of ontology (i.e. detection method, type of interaction, or biological database).

Each record in the *evidence* table contains a unique piece of supporting data for a given PPI, and includes annotation on the HVint2.0 identifier of the associated PPI that the evidence supports, protein identifiers of the source and target nodes before mapping to reference proteomes (i.e. as downloaded from the source databases, before homology or UniRef90 mapping), and PubMed identifiers of the primary citations. The data contained this table, in combination with the data on PPI detection method and type of interaction associated with each evidence record (retrievable through the *evidence_to_psimi* and *psimi_ontology* tables), are able to uniquely represent each piece of supporting evidence for each PPI. A total of 1739 evidence records are contained in the table.

Finally, the citation table contains details on the authorship, title, journal, publication year, PubMed and DOI identifiers, for the 106 citations in total, associated to the evidence records in the database.

All tables are annotated with the timestamp of the current update.
