# Supplementary material for: Conserved Central Intraviral Protein Interactome of the Herpesviridae Family
Source: mSystems. 2019 Oct 1;4(5):e00295-19. doi: 10.1128/mSystems.00295-19 (PMC6774017; doi:10.1128/mSystems.00295-19)
Supplement: TABLE S3 [file mSystems.00295-19-st003.docx]

| **Table S3.** | | |
| --- | --- | --- |
| **Protein** | **PDB identifier** | **Primary citation** |
| UL26 | 1AT3, 1CMV, 1FL1, 1ID4, 1IEC, 1IED, 1IEF, 1IEG, 1JQ6, 1JQ7, 1LAY, 1NJT, 1NJU, 1NKK, 1NKM, 1O6E, 1VZV, 1WPO, 2PBK, 3NJQ, 4CX8, 4P2T, 4P3H, 4V07, 4V08, 4V0T, 5UR3, 5UTE, 5UTN, 5UV3, 5UVP, 5V5D, 5V5E | 12421561, 9096314, 8805706, 17870089, 21723875, 26161660, 24977643, 26161660, 28759216 |
| UL30 | 1DML, 1T6L, 1YYP, 2GV9, 2Z0L | 15260974, 16371349, 16638752, 19801550 |
| UL42 | 5IWD, 5IXA | 28183184 |
| UL2 | 1LAU, 1UDG, 1UDH, 1UDI, 2C53, 2C56, 2J8X, 4L5N, 5AYS, 5NN7, 5NNH, 5NNU | 7845459, 7552746, 16306042, 17157317, 23892286, 26980279 |
| VP5 | 1NO7, 5VKU, 6B43, 6CGR | 12574112, 28663444, 29342139 |
| UL35 | 5VKU, 6N43, 6CGR | 28663444, 29342139 |
| UL18 | 5VKU, 6B43, 6CGR | 28663444, 29342139 |
| UL38 | 5VKU, 6B43, 6CGR | 28663444, 29342139 |
| UL29 | 1URJ | 15507432 |
| UL25 | 5F5U | 16474137 |
| UL27 | 2GUM, 3FVC, 3NW8, 3NWA, 3NWD, 3NWF, 4BOM, 4HSI, 4OTI, 5C6T, 5CXF, 5FZ2, 5YS2, 5YS6, 6ESC | 16840698, 19196955, 20943984, 23850455, 23500487, 25299639, 26365435, 26484870, 27035968, 29261802, |
| UL12 | 2W45, 2W4B | 19538972 |
| UL22/UL1 | 2XQY, 3M1C, 3PHF, 5T1D, 5VOC, 5VOB, 5VOB, 5W0K | 21149698, 20601960, 21149717, 27929061, 28783665, 28939750 |
| UL15 | 3N4P, 3N4Q,4IOX,5HUW | 20805464, 23596306, 27033706 |
| UL37 | 4K70, 5J2Z, 5VYL | 24599989, 29216315, 28768862 |
| UL36 | 4TT0, 4TT1 | 25678705 |
| UL54 | 4YXP, 5BQK | 26062451, 26085142 |
| UL31/UL34 | 4Z3U, 4ZXS, 5A3G, 5D5N, 5DOC, 5DOB, 5DOE, 5E8C, 5FKI | 26511020, 26150520, 26432641, 26511021, 26711332, 26711332 |
| UL21 | 5ED7 | 27053559 |
| UL28 | 5HUY | 27033706 |
