# Supplementary material for: Conserved Central Intraviral Protein Interactome of the Herpesviridae Family
Source: mSystems. 2019 Oct 1;4(5):e00295-19. doi: 10.1128/mSystems.00295-19 (PMC6774017; doi:10.1128/mSystems.00295-19)
Supplement: TABLE S4 [file mSystems.00295-19-st004.docx]

| **Table S4.** | | | | |
| --- | --- | --- | --- | --- |
| **Species** | **Protein (UniProtKB)** | **Gene name** | **Proposed biological roles** | **Original Source** |
| **HSV1** | O09802 | US8.5 | uncharacterised | UniProtKB,GO,18596102 |
| **HSV1** | P03176 | TK UL23 | replication | UniProtKB,GO,18596102 |
| **HSV1** | P04485 | ICP22 US1 | replication | UniProtKB,GO,18596102 |
| **HSV1** | P04487 | US11 | evasion or tolerance by virus of host immune response | UniProtKB,GO,18596102,1316472 |
| **HSV1** | P04488 | gE US8 | evasion or tolerance by virus of host immune response, tegumentation (secondary envelopment), envelopment | UniProtKB,GO,18596102 |
| **HSV1** | P06480 | gJ US5 | evasion or tolerance by virus of host immune response, tegumentation (secondary envelopment), envelopment | UniProtKB,GO,18596102 |
| **HSV1** | P06484 | gG US4 | evasion or tolerance by virus of host immune response, tegumentation (secondary envelopment), envelopment | UniProtKB,GO,18596102 |
| **HSV1** | P06485 | US2 | uncharacterised | UniProtKB,GO,18596102 |
| **HSV1** | P06486 | US10 | uncharacterised | UniProtKB,GO,18596102,9367380 |
| **HSV1** | P06492 | UL48 | tegumentation, transcription | UniProtKB,GO,18596102 |
| **HSV1** | P08392 | ICP4 IE175, RS1 | replication | UniProtKB,GO,18596102 |
| **HSV1** | P08393 | ICP0 IE110 | replication | UniProtKB,GO,18596102 |
| **HSV1** | P10187 | UL3 | uncharacterised | UniProtKB,GO,18596102 |
| **HSV1** | P10188 | UL4 | uncharacterised | UniProtKB,GO,18596102 |
| *… continues on the next page* | | | | |

| **Species** | **Protein (UniProtKB)** | **Gene name** | **Proposed biological roles** | **Original Source** |
| --- | --- | --- | --- | --- |
| **HSV1** | P10193 | UL9 | replication | UniProtKB,GO,18596102 |
| **HSV1** | P10204 | UL20 | tegumentation/egress | UniProtKB,GO,18596102,18434401 |
| **HSV1** | P10224 | RIR2 UL40 | replication | UniProtKB,GO,18596102 |
| **HSV1** | P10225 | UL41 | evasion or tolerance by virus of host immune response | UniProtKB,GO,18596102,11586916 |
| **HSV1** | P10227 | UL43 | uncharacterised | UniProtKB,GO,18596102,15780872,ViPR |
| **HSV1** | P10228 | gC UL44 | viral entry into host cell | UniProtKB,GO,18596102 |
| **HSV1** | P10229 | UL45 | evasion or tolerance by virus of host immune response, tegumentation (secondary envelopment), envelopment | UniProtKB,GO,18596102 |
| **HSV1** | P10230 | UL46 | tegumentation | UniProtKB,GO,18596102 |
| **HSV1** | P10231 | UL47 | tegumentation (primary envelopment, nuclear egress) | UniProtKB,GO,18596102 |
| **HSV1** | P10233 | UL49 | tegumentation | UniProtKB,GO,18596102 |
| **HSV1** | P10239 | UL55 | uncharacterised | UniProtKB,GO,18596102 |
| **HSV1** | P10240 | UL56 | uncharacterised | UniProtKB,GO,18596102,20682038 |
| **HSV1** | P68331 | gK UL53 | tegumentation (secondary envelopment) | UniProtKB,GO,25746217 |
| **HSV1** | Q69091 | gD US6 | viral entry into host cell | UniProtKB,GO,18596102 |
| *… continues on the next page* | | | | |

| **Species** | **Protein (UniProtKB)** | **Gene name** | **Proposed biological roles** | **Original Source** |
| --- | --- | --- | --- | --- |
| **HCMV** | C1BEG3 | UL74A | uncharacterised | UniProtKB |
| **HCMV** | F5H8S6 | US24 | replication | UniProt,15452216,16912288 |
| **HCMV** | F5H9N4 | UL24 | uncharacterised | UniProt,15452216 |
| **HCMV** | F5HAS7 | UL92 | replication | 24131715 |
| **HCMV** | F5HAZ3 | US23 | uncharacterised | UniProt |
| **HCMV** | F5HBC6 | UL82 | replication | InterPro,UniProt,15452216,18535146 |
| **HCMV** | F5HC71 | UL111A | evasion or tolerance by virus of host immune response | InterPro,UniProt |
| **HCMV** | F5HDC7 | US22 | uncharacterised | UniProt,15452216 |
| **HCMV** | F5HE12 | UL35 | tegumentation/replication | UniProtKB,15452216,11836424 |
| **HCMV** | F5HF62 | US28 | evasion or tolerance by virus of host immune response | InterPro,GOC,UniProt,19594424 |
| **HCMV** | F5HF90 | UL22A | uncharacterised | UniProt,15452216 |
| **HCMV** | F5HFJ8 | UL91 | replication | 23720731 |
| **HCMV** | F5HG98 | UL38 | evasion or tolerance by virus of host immune response | UniProtKB,15452216,18535146 |
| **HCMV** | F5HGG3 | UL26 | tegumentation/replication | UniProt,15452216,24505393 |
| **HCMV** | F5HGJ4 | UL25 | uncharacterised | UniProt,15452216 |
| **HCMV** | F5HGU6 | UL132 | uncharacterised | UniProt,15452216,16140760 |
| **HCMV** | Q6RJQ3 | UL141 | evasion or tolerance by virus of host immune response | UniProt,23498957 |
| **HCMV** | Q6SW29 | UL122 | replication | InterPro,UniProt,15452216 |
| **HCMV** | Q6SW37 | UL112/UL113 | replication | UniProtKB,GO,20538862 |
| **HCMV** | Q6SW59 | UL83 | evasion or tolerance by virus of host immune response | InterPro,UniProt,15452216 |
| **HCMV** | Q6SW89 | UL43 | uncharacterised | UniProt,15452216 |
| **HCMV** | Q6SW94 | UL37 | evasion or tolerance by virus of host immune response | UniProt,21177823 |
| **HCMV** | Q6SW99 | UL32 | tegumentation (secondary envelopment) | InterPro,UniProt,15452216,18653449 |
| *… continues on the next page* | | | | |

| **Species** | **Protein (UniProtKB)** | **Gene name** | **Proposed biological roles** | **Original Source** |
| --- | --- | --- | --- | --- |
| **EBV** | A0A1P7U1U3 | A73 | uncharacterised | UniProtKB,GO |
| **EBV** | P03177 | TK BXLF1 | replication | UniProtKB,GO |
| **EBV** | P03179 | BNRF1 | replication/dna packaging | UniProtKB,GO |
| **EBV** | P03180 | BCRF1 | evasion or tolerance by virus of host immune response | UniProtKB,GO |
| **EBV** | P03182 | BHRF1 | evasion or tolerance by virus of host immune response | UniProtKB,GO |
| **EBV** | P03192 | BMRF2 | viral entry into host cell | UniProtKB,GO |
| **EBV** | P03197 | BLRF2 | viral spread/tegumentation | UniProtKB,GO,23326445 |
| **EBV** | P03199 | BLLF2 | uncharacterised | UniProtKB,GO |
| **EBV** | P03205 | BZLF2 | viral entry into host cell | UniProtKB,GO |
| **EBV** | P03206 | BZLF1 | regulation of transcription, evasion or tolerance by virus of host immune response | UniProtKB,GO,20080764,15464842 |
| **EBV** | P03207 | BRRF1 | regulation of transcription | UniProtKB,GO,15113878 |
| **EBV** | P03208 | BILF1 | evasion or tolerance by virus of host immune response | UniProtKB,GO,23315076 |
| **EBV** | P03209 | BRLF1 | regulation of transcription | UniProtKB,GO |
| **EBV** | P03210 | BRRF2 | uncharacterised | UniProtKB,GO |
| **EBV** | P03224 | BDLF3 | evasion or tolerance by virus of host immune response | UniProtKB,GO,27077376 |
| **EBV** | P03225 | BDLF2 | viral spread | UniProtKB,GO,18995876 |
| **EBV** | P03228 | BARF1 | evasion or tolerance by virus of host immune response | UniProtKB,GO,18533018 |
| **EBV** | P03230 | LMP1 BNLF1 | evasion or tolerance by virus of host immune response | UniProtKB,GO |
| **EBV** | P0C739 | BNLF2a | evasion or tolerance by virus of host immune response | UniProtKB,GO |
| **EBV** | P0CAP6 | RIR2 BaRF1 | replication | UniprotKB,GO |
| **EBV** | P0CK56 | BDLF4 | uncharacterised | UniProtKB,GO |
| **EBV** | P0CK58 | BALF1 | evasion or tolerance by virus of host immune response | UniProtKB,GO,11836425,16277553,26468525 |
| *… continues on the next page* | | | | |

| **Species** | **Protein (UniProtKB)** | **Gene name** | **Proposed biological roles** | **Original Source** |
| --- | --- | --- | --- | --- |
| **EBV** | P13285 | LMP2 | evasion or tolerance by virus of host immune response | UniProtKB,GO |
| **EBV** | P30117 | BKRF4 | uncharacterised | UniprotKB,GO |
| **EBV** | Q8AZJ3 | BNLF2b | uncharacterised | UniProtKB,GO |
| **EBV** | Q8AZJ4 | BARF0 | uncharacterised | UniProtKB,GO |
| **EBV** | Q8AZK7 | EBNA-LP EBNA5 | regulation of transcription | UniProtKB,GO |
|  | | | | |
